# Crystal structure of 4-benzyl-2*H*-benzo[*b*][1,4]thia­zin-3(4*H*)-one

**DOI:** 10.1107/S2056989015022276

**Published:** 2015-11-28

**Authors:** N. K. Sebbar, M. Ellouz, E. M. Essassi, Y. Ouzidan, J. T. Mague

**Affiliations:** a Laboratoire de Chimie Organique Hétérocyclique URAC 21, Pôle de Compétence Pharmacochimie, Av. Ibn Battouta, BP 1014, Faculté des Sciences, Université Mohammed V, Rabat, Morocco; b Laboratoire de Chimie Organique Appliquée, Université Sidi Mohamed Ben Abdallah, Faculté des Sciences et Techniques, Route d’immouzzer, BP 2202, Fez, Morocco; c Department of Chemistry, Tulane University, New Orleans, LA 70118, USA

**Keywords:** crystal structure, 1,4-benzo­thia­zine derivatives, C—H⋯O inter­actions

## Abstract

In the title compound, C_15_H_13_NOS, the thia­zine ring adopts a twisted boat conformation and the dihedral angle between the aromatic rings is 86.54 (4)°. In the crystal, mol­ecules are linked by weak C—H⋯O inter­actions, resulting in chains along [010].

## Related literature   

For related structures and background to 1,4-benzo­thia­zine derivatives, see: Zerzouf *et al.* (2001 ▸); Sebbar *et al.* (2015 ▸).
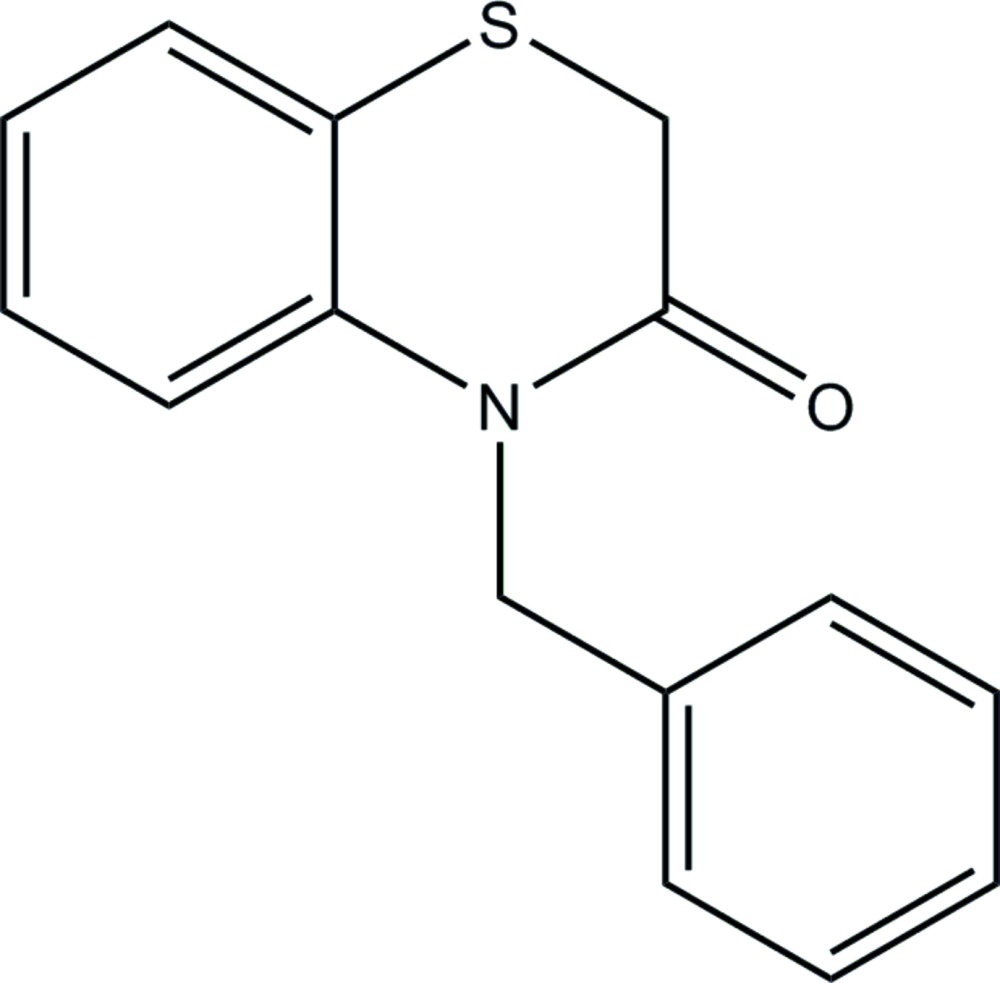


## Experimental   

### Crystal data  

C_15_H_13_NOS
*M* *_r_* = 255.32
Monoclinic, 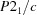

*a* = 10.8711 (7) Å
*b* = 5.3815 (3) Å
*c* = 21.1997 (13) Å
β = 93.128 (1)°
*V* = 1238.39 (13) Å^3^
*Z* = 4
Mo *K*α radiation
μ = 0.25 mm^−1^
*T* = 150 K
0.31 × 0.19 × 0.15 mm

### Data collection  

Bruker SMART APEX CCD diffractometer
Absorption correction: multi-scan (*SADABS*; Bruker, 2015 ▸) *T*_min_ = 0.88, *T*_max_ = 0.96
22588 measured reflections
3318 independent reflections
2731 reflections with *I* > 2σ(*I*)
*R*_int_ = 0.034

### Refinement  

*R*[*F* ^2^ > 2σ(*F* ^2^)] = 0.038
*wR*(*F* ^2^) = 0.107
*S* = 1.06
3318 reflections
163 parameters
H-atom parameters constrained
Δρ_max_ = 0.43 e Å^−3^
Δρ_min_ = −0.19 e Å^−3^

### 

Data collection: *APEX2* (Bruker, 2015 ▸); cell refinement: *SAINT* (Bruker, 2015 ▸); data reduction: *SAINT*; program(s) used to solve structure: *SHELXT* (Sheldrick, 2015*a* ▸); program(s) used to refine structure: *SHELXL2014* (Sheldrick, 2015*b* ▸); molecular graphics: *DIAMOND* (Brandenburg & Putz, 2012 ▸); software used to prepare material for publication: *SHELXTL* (Sheldrick, 2015*b* ▸).

## Supplementary Material

Crystal structure: contains datablock(s) global, I. DOI: 10.1107/S2056989015022276/hb7545sup1.cif

Structure factors: contains datablock(s) I. DOI: 10.1107/S2056989015022276/hb7545Isup2.hkl

Supporting information file. DOI: 10.1107/S2056989015022276/hb7545Isup3.cml

**Fig. 1.**
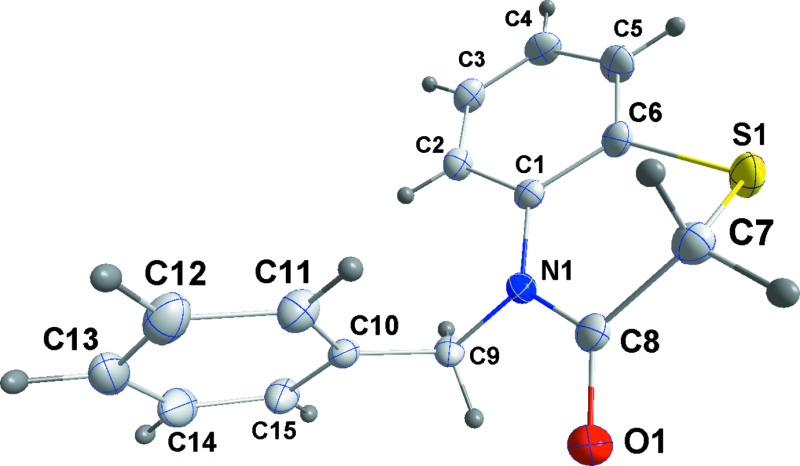
Perspective view of the molecule with 50% probability ellipsoids.

**Fig. 2.**
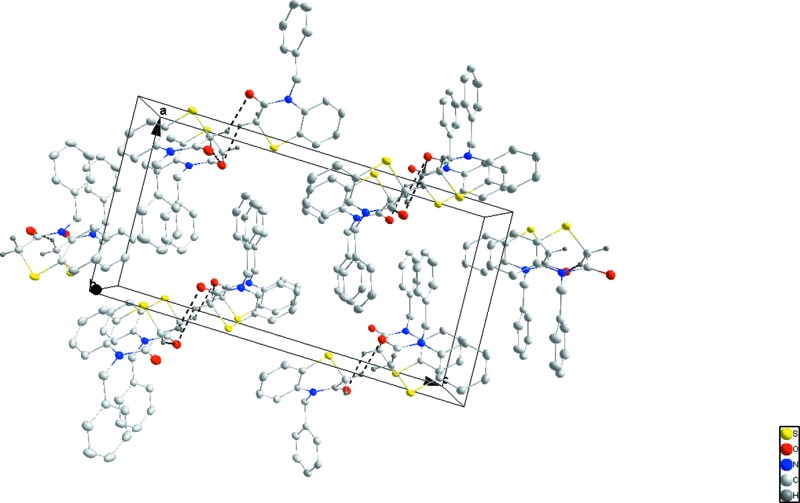
Packing viewed down the b axis. Intermolecular C—H···O interactions are shown by dotted lines.

. DOI: 10.1107/S2056989015022276/hb7545fig1.tif
Perspective view of the mol­ecule with 50% probability ellipsoids.

b . DOI: 10.1107/S2056989015022276/hb7545fig2.tif
Packing viewed down the *b* axis. Inter­molecular C—H⋯O inter­actions are shown by dotted lines.

CCDC reference: 1438105

Additional supporting information:  crystallographic information; 3D view; checkCIF report

## Figures and Tables

**Table 1:** Hydrogen-bond geometry (Å, °)

*D*—H⋯*A*	*D*—H	H⋯*A*	*D*⋯*A*	*D*—H⋯*A*
C7—H7*A*⋯O1^i^	0.99	2.55	3.2504 (14)	128
C7—H7*B*⋯O1^ii^	0.99	2.53	3.4403 (15)	152

Symmetry codes: (i) 
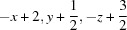
; (ii) 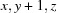.

## supplementary crystallographic information

### S1. Comment

As a continuation of our research devoted to the development of substituted 1,4-benzothiazine derivatives (Zerzouf *et al.*, 2001; Sebbar *et al.*, 2015), we report the synthesis of a 1,4-benzothiazine derivative by reaction of benzyl chloride with 2*H*-benzo[*b*][1,4]thiazin-3(4*H*)-one in the presence of tetra-*n*-butylammonium bromide as catalyst and potassium carbonate as base (Scheme 1).

In the title compound, the heterocyclic ring has puckering parameters Q = 0.6272 (10) Å, θ = 63.91 (10)° and φ = 325.56 (11)°. The dihedral angle between the rings C1—C6 and C10—C15 is 86.54 (4)°. Weak C—H···O interactions (Table 1) form chains running parallel to the *b* axis (Fig. 2).

### S2. Experimental

To a solution of 2*H*-benzo[*b*][1,4]thiazin-3(4*H*)-one (0.543 g, 3.29 mmol), benzyl chloride (0.76 ml, 6.58 mmol) and potassium carbonate (0.91 g, 6.58 mmol) in DMF (15 ml) was added a catalytic amount of tetra- *n*-butylammonium bromide (0.11 g, 0.33 mmol) and the mixture was stirred for 24 h. The solid material was removed by filtration and the solvent evaporated under vacuum. The solid product was purified by recrystallization from ethanol to afford colorless crystals in 75% yield.

### S3. Refinement

H-atoms attached to carbon were placed in calculated positions (C—H = 0.95 − 0.99 Å). All were included as riding contributions with isotropic displacement parameters 1.2 times those of the attached atoms.

### Crystal data

**Table:**

C_15_H_13_NOS	*F*(000) = 536
*M**_r_* = 255.32	*D*_x_ = 1.369 Mg m^−^^3^
Monoclinic, *P*2_1_/*c*	Mo *K*α radiation, λ = 0.71073 Å
*a* = 10.8711 (7) Å	Cell parameters from 9273 reflections
*b* = 5.3815 (3) Å	θ = 2.6–29.0°
*c* = 21.1997 (13) Å	µ = 0.25 mm^−^^1^
β = 93.128 (1)°	*T* = 150 K
*V* = 1238.39 (13) Å^3^	Block, colourless
*Z* = 4	0.31 × 0.19 × 0.15 mm

### Data collection

**Table:**

Bruker SMART APEX CCD diffractometer	3318 independent reflections
Radiation source: fine-focus sealed tube	2731 reflections with *I* > 2σ(*I*)
Graphite monochromator	*R*_int_ = 0.034
Detector resolution: 8.3333 pixels mm^-1^	θ_max_ = 29.1°, θ_min_ = 1.9°
φ and ω scans	*h* = −14→14
Absorption correction: multi-scan (*SADABS*; Bruker, 2015)	*k* = −7→7
*T*_min_ = 0.88, *T*_max_ = 0.96	*l* = −28→28
22588 measured reflections	

### Refinement

**Table:**

Refinement on *F*^2^	Primary atom site location: structure-invariant direct methods
Least-squares matrix: full	Secondary atom site location: difference Fourier map
*R*[*F*^2^ > 2σ(*F*^2^)] = 0.038	Hydrogen site location: inferred from neighbouring sites
*wR*(*F*^2^) = 0.107	H-atom parameters constrained
*S* = 1.06	*w* = 1/[σ^2^(*F*_o_^2^) + (0.0711*P*)^2^ + 0.0734*P*] where *P* = (*F*_o_^2^ + 2*F*_c_^2^)/3
3318 reflections	(Δ/σ)_max_ = 0.001
163 parameters	Δρ_max_ = 0.43 e Å^−^^3^
0 restraints	Δρ_min_ = −0.19 e Å^−^^3^

### Special details

**Table:**

Experimental. The diffraction data were obtained from 3 sets of 400 frames, each of width 0.5° in ω, colllected at φ = 0.00, 90.00 and 180.00° and 2 sets of 800 frames, each of width 0.45° in φ, collected at ω = −30.00 and 210.00°. The scan time was 20 sec/frame.
Geometry. All e.s.d.'s (except the e.s.d. in the dihedral angle between two l.s. planes) are estimated using the full covariance matrix. The cell e.s.d.'s are taken into account individually in the estimation of e.s.d.'s in distances, angles and torsion angles; correlations between e.s.d.'s in cell parameters are only used when they are defined by crystal symmetry. An approximate (isotropic) treatment of cell e.s.d.'s is used for estimating e.s.d.'s involving l.s. planes.
Refinement. Refinement of *F*^2^ against ALL reflections. The weighted *R*-factor *wR* and goodness of fit *S* are based on *F*^2^, conventional *R*-factors *R* are based on *F*, with *F* set to zero for negative *F*^2^. The threshold expression of *F*^2^ > σ(*F*^2^) is used only for calculating *R*-factors(gt) *etc*. and is not relevant to the choice of reflections for refinement. *R*-factors based on *F*^2^ are statistically about twice as large as those based on *F*, and *R*- factors based on ALL data will be even larger. H-atoms attached to carbon were placed in calculated positions (C—H = 0.95 − 0.99 Å). All were included as riding contributions with isotropic displacement parameters 1.2 times those of the attached atoms.

### Fractional atomic coordinates and isotropic or equivalent isotropic displacement parameters (Å^2^)

**Table:**

	*x*	*y*	*z*	*U*_iso_*/*U*_eq_	
S1	1.01122 (3)	0.51867 (6)	0.64271 (2)	0.02520 (11)	
O1	0.82014 (8)	0.06255 (17)	0.72781 (4)	0.0263 (2)	
N1	0.77044 (9)	0.22455 (18)	0.63087 (4)	0.0188 (2)	
C1	0.78669 (10)	0.4044 (2)	0.58307 (5)	0.0183 (2)	
C2	0.69629 (11)	0.4345 (2)	0.53375 (5)	0.0228 (2)	
H2	0.6216	0.3430	0.5342	0.027*	
C3	0.71505 (12)	0.5966 (2)	0.48439 (5)	0.0266 (3)	
H3	0.6537	0.6136	0.4510	0.032*	
C4	0.82274 (12)	0.7341 (2)	0.48348 (5)	0.0283 (3)	
H4	0.8359	0.8435	0.4493	0.034*	
C5	0.91096 (12)	0.7109 (3)	0.53267 (5)	0.0273 (3)	
H5	0.9839	0.8080	0.5325	0.033*	
C6	0.89443 (10)	0.5470 (2)	0.58242 (5)	0.0204 (2)	
C7	0.90903 (11)	0.4571 (2)	0.70494 (5)	0.0202 (2)	
H7A	0.9579	0.4299	0.7451	0.024*	
H7B	0.8549	0.6026	0.7104	0.024*	
C8	0.83135 (10)	0.2310 (2)	0.68959 (5)	0.0191 (2)	
C9	0.68427 (11)	0.0192 (2)	0.61862 (6)	0.0206 (2)	
H9A	0.7166	−0.1296	0.6415	0.025*	
H9B	0.6814	−0.0193	0.5729	0.025*	
C10	0.55414 (10)	0.0666 (2)	0.63773 (5)	0.0185 (2)	
C11	0.52211 (11)	0.2662 (2)	0.67490 (5)	0.0239 (3)	
H11	0.5828	0.3854	0.6879	0.029*	
C12	0.40231 (12)	0.2935 (3)	0.69330 (5)	0.0290 (3)	
H12	0.3813	0.4312	0.7186	0.035*	
C13	0.31340 (12)	0.1201 (3)	0.67478 (6)	0.0302 (3)	
H13	0.2314	0.1387	0.6874	0.036*	
C14	0.34421 (12)	−0.0800 (3)	0.63797 (7)	0.0319 (3)	
H14	0.2835	−0.1998	0.6255	0.038*	
C15	0.46353 (11)	−0.1061 (2)	0.61924 (6)	0.0261 (3)	
H15	0.4839	−0.2430	0.5935	0.031*	

### Atomic displacement parameters (Å^2^)

**Table:**

	*U*^11^	*U*^22^	*U*^33^	*U*^12^	*U*^13^	*U*^23^
S1	0.01580 (17)	0.0344 (2)	0.02528 (17)	−0.00305 (11)	−0.00034 (12)	0.00452 (11)
O1	0.0243 (4)	0.0259 (5)	0.0282 (4)	−0.0015 (4)	−0.0014 (3)	0.0087 (3)
N1	0.0177 (4)	0.0169 (5)	0.0215 (4)	−0.0014 (4)	−0.0006 (3)	−0.0006 (3)
C1	0.0192 (5)	0.0181 (5)	0.0178 (5)	0.0024 (4)	0.0019 (4)	−0.0025 (4)
C2	0.0227 (6)	0.0244 (6)	0.0210 (5)	−0.0001 (5)	−0.0022 (4)	−0.0040 (4)
C3	0.0296 (6)	0.0309 (7)	0.0189 (5)	0.0040 (5)	−0.0033 (5)	−0.0025 (5)
C4	0.0343 (7)	0.0302 (7)	0.0209 (5)	0.0020 (5)	0.0051 (5)	0.0054 (5)
C5	0.0243 (6)	0.0318 (7)	0.0263 (6)	−0.0026 (5)	0.0060 (5)	0.0039 (5)
C6	0.0171 (5)	0.0245 (6)	0.0198 (5)	0.0022 (4)	0.0019 (4)	−0.0011 (4)
C7	0.0200 (5)	0.0219 (6)	0.0186 (5)	−0.0010 (4)	−0.0006 (4)	0.0009 (4)
C8	0.0157 (5)	0.0195 (6)	0.0220 (5)	0.0034 (4)	0.0011 (4)	0.0006 (4)
C9	0.0195 (6)	0.0145 (5)	0.0276 (6)	−0.0002 (4)	0.0003 (4)	−0.0032 (4)
C10	0.0187 (5)	0.0167 (5)	0.0200 (5)	0.0008 (4)	−0.0010 (4)	0.0021 (4)
C11	0.0239 (6)	0.0234 (6)	0.0242 (5)	0.0017 (5)	−0.0014 (4)	−0.0022 (4)
C12	0.0302 (7)	0.0345 (7)	0.0228 (5)	0.0097 (6)	0.0050 (5)	−0.0008 (5)
C13	0.0218 (6)	0.0410 (8)	0.0283 (6)	0.0048 (5)	0.0055 (5)	0.0115 (6)
C14	0.0218 (6)	0.0306 (7)	0.0430 (7)	−0.0051 (5)	−0.0014 (5)	0.0061 (6)
C15	0.0236 (6)	0.0196 (6)	0.0349 (6)	−0.0012 (5)	−0.0004 (5)	−0.0023 (5)

### Geometric parameters (Å, º)

**Table:**

S1—C6	1.7583 (11)	C7—H7A	0.9900
S1—C7	1.8013 (12)	C7—H7B	0.9900
O1—C8	1.2262 (14)	C9—C10	1.5144 (16)
N1—C8	1.3781 (13)	C9—H9A	0.9900
N1—C1	1.4191 (14)	C9—H9B	0.9900
N1—C9	1.4628 (14)	C10—C11	1.3880 (16)
C1—C6	1.4012 (16)	C10—C15	1.3947 (16)
C1—C2	1.4047 (15)	C11—C12	1.3876 (17)
C2—C3	1.3860 (18)	C11—H11	0.9500
C2—H2	0.9500	C12—C13	1.3848 (19)
C3—C4	1.3859 (19)	C12—H12	0.9500
C3—H3	0.9500	C13—C14	1.382 (2)
C4—C5	1.3833 (16)	C13—H13	0.9500
C4—H4	0.9500	C14—C15	1.3843 (18)
C5—C6	1.3940 (17)	C14—H14	0.9500
C5—H5	0.9500	C15—H15	0.9500
C7—C8	1.5066 (16)		
			
C6—S1—C7	95.66 (5)	O1—C8—N1	121.21 (10)
C8—N1—C1	123.74 (9)	O1—C8—C7	121.93 (10)
C8—N1—C9	116.79 (9)	N1—C8—C7	116.84 (9)
C1—N1—C9	119.46 (9)	N1—C9—C10	115.07 (9)
C6—C1—C2	118.72 (10)	N1—C9—H9A	108.5
C6—C1—N1	121.15 (9)	C10—C9—H9A	108.5
C2—C1—N1	120.07 (10)	N1—C9—H9B	108.5
C3—C2—C1	120.60 (11)	C10—C9—H9B	108.5
C3—C2—H2	119.7	H9A—C9—H9B	107.5
C1—C2—H2	119.7	C11—C10—C15	118.70 (11)
C4—C3—C2	120.38 (11)	C11—C10—C9	123.32 (10)
C4—C3—H3	119.8	C15—C10—C9	117.92 (10)
C2—C3—H3	119.8	C12—C11—C10	120.65 (12)
C5—C4—C3	119.52 (11)	C12—C11—H11	119.7
C5—C4—H4	120.2	C10—C11—H11	119.7
C3—C4—H4	120.2	C13—C12—C11	120.02 (12)
C4—C5—C6	121.00 (12)	C13—C12—H12	120.0
C4—C5—H5	119.5	C11—C12—H12	120.0
C6—C5—H5	119.5	C14—C13—C12	119.89 (12)
C5—C6—C1	119.76 (10)	C14—C13—H13	120.1
C5—C6—S1	119.15 (9)	C12—C13—H13	120.1
C1—C6—S1	121.08 (9)	C13—C14—C15	120.04 (12)
C8—C7—S1	110.56 (8)	C13—C14—H14	120.0
C8—C7—H7A	109.5	C15—C14—H14	120.0
S1—C7—H7A	109.5	C14—C15—C10	120.69 (12)
C8—C7—H7B	109.5	C14—C15—H15	119.7
S1—C7—H7B	109.5	C10—C15—H15	119.7
H7A—C7—H7B	108.1		
			
C8—N1—C1—C6	23.43 (16)	C1—N1—C8—O1	−175.23 (10)
C9—N1—C1—C6	−156.89 (11)	C9—N1—C8—O1	5.09 (15)
C8—N1—C1—C2	−159.32 (11)	C1—N1—C8—C7	6.40 (15)
C9—N1—C1—C2	20.36 (15)	C9—N1—C8—C7	−173.29 (10)
C6—C1—C2—C3	1.99 (17)	S1—C7—C8—O1	131.04 (10)
N1—C1—C2—C3	−175.32 (11)	S1—C7—C8—N1	−50.60 (12)
C1—C2—C3—C4	−0.93 (19)	C8—N1—C9—C10	88.08 (12)
C2—C3—C4—C5	−0.81 (19)	C1—N1—C9—C10	−91.62 (12)
C3—C4—C5—C6	1.48 (19)	N1—C9—C10—C11	−12.01 (16)
C4—C5—C6—C1	−0.40 (19)	N1—C9—C10—C15	170.98 (10)
C4—C5—C6—S1	178.35 (10)	C15—C10—C11—C12	0.00 (17)
C2—C1—C6—C5	−1.33 (17)	C9—C10—C11—C12	−176.99 (11)
N1—C1—C6—C5	175.96 (11)	C10—C11—C12—C13	0.24 (18)
C2—C1—C6—S1	179.95 (9)	C11—C12—C13—C14	0.01 (18)
N1—C1—C6—S1	−2.77 (15)	C12—C13—C14—C15	−0.51 (19)
C7—S1—C6—C5	147.46 (11)	C13—C14—C15—C10	0.75 (19)
C7—S1—C6—C1	−33.80 (10)	C11—C10—C15—C14	−0.49 (18)
C6—S1—C7—C8	57.87 (9)	C9—C10—C15—C14	176.66 (11)

### Hydrogen-bond geometry (Å, º)

**Table:**

*D*—H···*A*	*D*—H	H···*A*	*D*···*A*	*D*—H···*A*
C7—H7*A*···O1^i^	0.99	2.55	3.2504 (14)	128
C7—H7*B*···O1^ii^	0.99	2.53	3.4403 (15)	152

Symmetry codes: (i) −*x*+2, *y*+1/2, −*z*+3/2; (ii) *x*, *y*+1, *z*.
