# Supplementary figures and images for: Crystal structure of 4-benzyl-2H-benzo[b][1,4]thia­zin-3(4H)-one
Source: Acta Crystallogr E Crystallogr Commun. 2015 Nov 28;71(Pt 12):o999. doi: 10.1107/S2056989015022276 (PMC4719943; doi:10.1107/S2056989015022276)

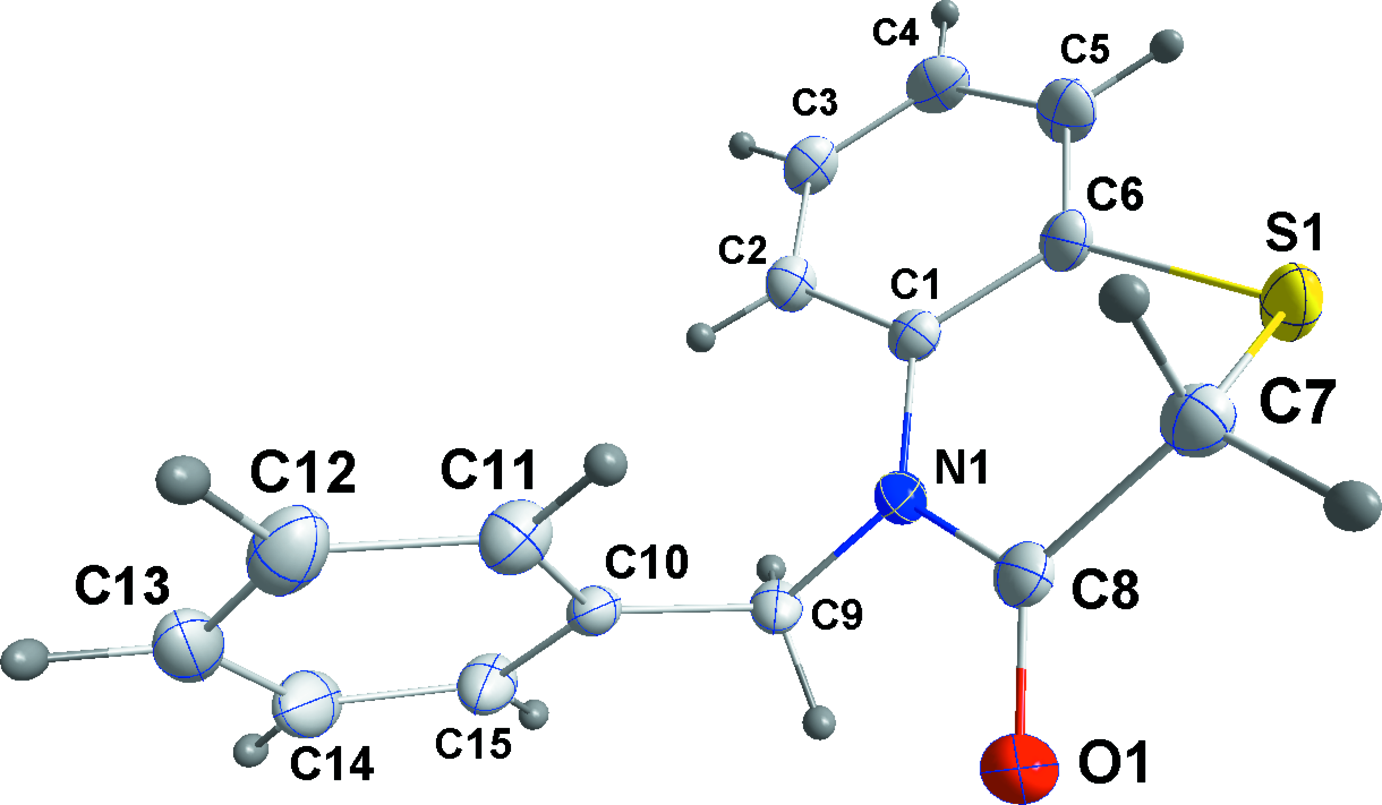

Supplement: Supplementary file 4 [file e-71-0o999-fig1.tif]

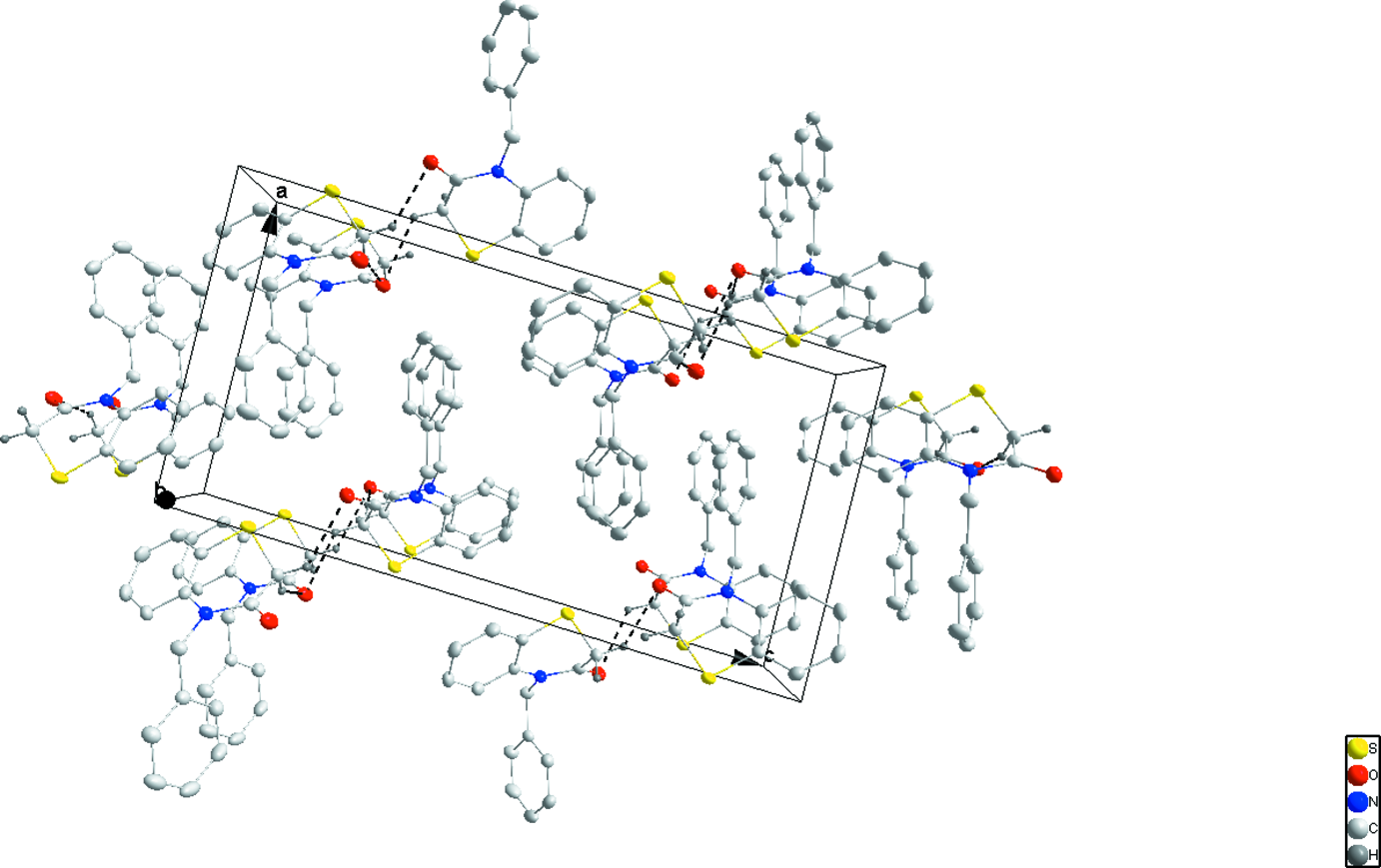

Supplement: Supplementary file 5 [file e-71-0o999-fig2.tif]
